# ‘The Medical’ and ‘Health’ in a Critical Medical Humanities

**DOI:** 10.1007/s10912-014-9314-4

**Published:** 2014-12-12

**Authors:** Sarah Atkinson, Bethan Evans, Angela Woods, Robin Kearns

**Affiliations:** 1 Department of Geography and the Centre for Medical Humanities, Durham University, Durham, DH1 3LE UK; 2 Department of Geography and Planning, University of Liverpool, Liverpool, L69 7ZQ UK; 3 The Centre for Medical Humanities, Durham University, Caedmon Building, Leazes Rd., Durham, DH1 1SZ UK; 4 School of Environment, The University of Auckland, Private Bag 92019, Auckland, New Zealand

**Keywords:** Medical humanities, Health humanities, Medical geography, Geographies of health, Critical theory, Arts and health, Activism, Patient advocacy

## Abstract

As befits an emerging field of enquiry, there is on-going discussion about the scope, role and future of the medical humanities. One relatively recent contribution to this debate proposes a differentiation of the field into two distinct terrains, ‘medical humanities’ and ‘health humanities,’ and calls for a supersession of the former by the latter. In this paper, we revisit the conceptual underpinnings for a distinction between ‘the medical’ and ‘health’ by looking at the history of an analogous debate between ‘medical geography’ and ‘the geographies of health’ that has, over the last few years, witnessed a re-blurring of the distinction. Highlighting the value of this debate within the social sciences for the future development of the medical humanities, we call for scholars to take seriously the challenges of critical and cultural theory, community-based arts and health, and the counter-cultural creative practices and strategies of activist movements in order to meet the new research challenges and fulfill the radical potential of a critical medical humanities.

## Under scrutiny: medical humanities and health

In the last 10 years, the number of disciplines, subjects, methodologies and individual researchers and practitioners working or associated with the medical humanities has grown considerably. As befits an emerging field of enquiry, there is on-going debate about its future direction (Ahlzén 2007; Evans and Greaves 2003, 2010; Evans and Macnaughton 2004; Macnaughton 2011; Pattison 2003), complicated by the fact that it “is an area of scholarship, education and creativity peopled with those who primarily, secondarily or in no way associate themselves with the field” (Shafer 2009, 3). Tracing its roots to medical education – and to a territory also fertilized by medical history and bioethics – the medical humanities are most frequently thought of as a means of mobilizing the arts and humanities in the context of medical practice and pedagogy. However, contemporary medical humanities research, while having its focus firmly on matters medical, broadly construed, is not calculated exclusively towards improving the *practice* of healthcare, but also, crucially, on better understanding its conceptualization and representation (Carel and Cooper 2012), its regulation through government and other policies (Metzl and Kirkland 2009), its history (Foucault 1994; 2002), and the complex ways in which cultures influence and are influenced by “medicalized” accounts of human endeavour (Murray 2008; Woods 2011). Despite claims that the medical humanities has become a “mature discipline” (Crawford et al. 2010), this recent upsurge of interest and engagement with an increasingly diverse range of topics we think speaks to the youthful, exploratory vigour of a field which is better thought of as “beginning maturation” (Ahlzén 2007). We use the world “field” advisedly, for as well as being not (yet) mature, the medical humanities is also, and very self-consciously, *not* a discipline. Whether it is multidisciplinary (Chambers 2009), interdisciplinary (Evans and Macnaughton 2004), or postdisciplinary (Lewis 1998) is still and perhaps necessarily debated, but as Stephen Pattison has noted, “We will know that medical humanities as a vibrant, pluralistic, experimental, risky movement has died when,” among other things, it “excludes varieties of disciplinary perspective and performance and becomes an autonomous discipline in its own right.” (2003, 34).

One of the most widely discussed contributions to these debates about disciplinary purpose and identity has been a call by Paul Crawford and colleagues for a move from “medical” to “health” humanities. Central to their argument is the claim that the medical humanities have an overly narrow focus on “the medical” and marginalize the experiences of allied health professionals, nurses, carers and patients. Such a view is, we suggest, empirically and conceptually questionable. While it seems plausible to suggest that the clinical encounter does retain a privileged place in the medical humanities imaginary, the idea that the field has worked to exclude any voices but those of the doctor or medical student is clearly false. Some of the best work in the medical humanities has sought to examine not just the practice of healthcare in a diverse range of institutional and social contexts (Colls and Evans 2008), but also to consider ‘medicalization’ itself as a pervasive cultural force, and to question the historical and contemporary expansion of a ‘medical’ gaze (see for example, Coors 2003; Heath 2010). Furthermore, understanding the subjective experience of illness as something distinct from the biomedical attribution of disease is an enterprise uniting most, if not all, medical humanities scholars. The idea that the individual’s or patient’s perspective is somehow absent is therefore difficult to support. The argument, then, for a new discipline of ‘health humanities’ which is “more inclusive, outward-facing and applied” and would “engage with the contributions of those marginalized from the medical humanities” (Crawford et al. 2010, 4) is premised on a misleadingly narrow view of the field’s existing scope and depth. Moreover, the implication of a robust distinction between the two “disciplines” is also misleading; the health humanities, as described, promises an expanded programme of research and practice only in relation to a small fraction of the work that is currently pursued under the umbrella of medical humanities. The proposition, then, is not about a new perspective or broadening the theoretical or philosophical questions asked by the medical humanities, but simply about reaching out to include those “non-medical” health professions perceived to have been excluded. This not only fails to recognise much of the work published and presented in medical humanities arenas (see for example Davis 2005; Flaming 2005; Nestel 1998), but more worryingly, completely bypasses a critical engagement with different understandings of what is meant by the key concepts of “the medical” and of “health”, a critical engagement that has been characteristic of research within the social sciences for at least two decades.

This kind of critical engagement is, we suggest, far more fundamental to any evolution of the medical humanities. Critical engagements with “the medical” which open out and interrogate the multiple ways in which “the medical,” medicine and health are encountered and experienced are not only important and desirable in their own right, as we will show, but would also facilitate recognition of the breadth and vibrancy of medical humanities research without the need to draw disciplinary lines around particular types of work. In order to advance such critical engagement, and in the context of this special issue, we will draw on the debates within one social science field, that of human geography, from the late 1990s and into the early 2000s. A “critical” geography specifically attends to the situated nature of health and health practices in both space and time, and as such challenges conventional treatments of context as either backdrop or determinant. So it is from “critical” geography, we suggest, that the medical humanities can draw their inspiration without jeopardizing the openness and heterogeneity of the field.

## Critical distinctions: medical geography and geographies of health

“Medical geography,” as a sub-discipline of human geography, has historically been dominated by epidemiological frameworks and biomedical models of health and illness. As such, much of medical geography prior to the past two decades sought to identify variations in the spatial prevalence of disease and to differentiate the contextual/environmental and compositional/individual determinants of ill health (for example, Cliff et al. 1981; Cliff and Haggett 1985; Learmonth 1988; Thomas 1992). However, in the ‘nineties, the field of human geography, along with other social sciences, embraced a cultural and critical turn which, within the sub-field of “medical geography,” led to a series of exchanges between those who wanted to “preserve” research on health, disease and illness as a conventionally spatial science and those interested in expanding the theoretical, empirical and political mode of inquiry (Andrews et al. 2012; Kearns and Collins 2010) New labels of “medical” geography and geographies of “health” initially served to distinguish different approaches, a distinction which both reflected and advanced contemporary movements within medical practice and public health policy. From its start, the World Health Organization enshrined in its constitution a vision of health as more than the absence of disease (WHO 1946). This vision was operationalized first through the Declaration of Alma Ata and its policy emphasis on primary health care, the prevention of disease and attention to wider determinants of ill-health (WHO 1978) and later through the Ottawa Charter and the call for a “new public health” directed to health promotion (WHO 1986). The distinction made between a clinically oriented medicine, or biomedicine, and a politically oriented social medicine, or public health, was picked up and elaborated within the social sciences through both theoretical and critical engagements.

Within geography, the distinction between “the medical” and “health” was expressed through three important areas of contention. The first of these concerned the object of study. Critics of a medical geography that was focussed on a largely descriptive spatial epidemiology sought to challenge and extend the focus of the sub-discipline by adopting the WHO conception of health, seen not merely as the absence of disease, but as related to broader definitions of well-being, inequity and social justice (Kearns 1993; Kearns and Moon 2002; Moss and Dyck 1996; Smith 1973; Smith 1994; Smith et al. 2003). Researchers also directed attention to the processes and relations between places, health and health care, for example, through developing the concept of therapeutic spaces and landscapes (Conradson 2005; Gesler 1992, 2000; Smyth 2005; Tonnellier and Curtis 2005; Williams 2007) or by giving value to experiential and emotionally inflected understandings of such relations (Anderson and Smith 2001; Atkinson and Farias 1995; Davidson et al. 2005; Dyck 1992; 2003; Gesler and Kearns 2002; Milligan 2003; 2005). Moreover, from the ‘nineties onwards, the social sciences in general took far greater interest in the body itself as the focus of enquiry, a move eagerly embraced by geographers who saw the theoretical possibilities in the body as site, or as poet Adrienne Rich put it, ‘the geography closest in’ (Rich 1986, 212; Simonsen 2000; Longhurst 1995; 1997). This new focus on the body by geographers brought a second point of contention around the place for critical theory. Rather than treating bodies as ‘dots on a map’, neither embodied, reflexive nor agentive, feminist and poststructuralist geographers demonstrated the need to question the social meanings attributed to particular forms of embodiment and, by engaging with texts such as Foucault’s *Birth of the Clinic* (1994), to analyse the spatialities of power inherent in medical engagements with particular bodies (Foucault 1991; Longhurst 2000; Miller and Rose 2008; Parr 2002). Finally, debate focussed on new methodologies and epistemologies. How should geographers *do* this more critical work? Mapping incidences of disease, no matter how rigorous the statistical analysis, does not in itself further an understanding of the cultural specificity of embodiment, well-being, or the politics of health. What kind of knowledge should geographers pursue and with what imagined consequences and effects? Here, a divide emerged between those who positioned themselves as “policy-relevant” medical geographers through arguing that “real-world” and often quantitative data-sets were necessary to speak to and make an impact on policy (Dorling and Shaw 2002; Kearns and Moon 2002) and those who asserted that the more theoretical, emerging critical health geographies were important (Parr 2002) since “critical work which questions and contests the categories used in biomedical science has a clear and important role to play in medical geography’s engagement with policy and debates around inequalities in health and health care – highlighting the processes by which some bodies are seen as more equal than others” (Evans 2006, 260).

These debates map on to structural and lingering issues in the medical humanities, particularly with regard to the definition and status of “the medical.” Although it retains the name, the medical humanities, particularly in research terms, shares many of the concerns of the “new” medical, post-medical or health geography. These include rejecting biomedical reductionism without abandoning the materiality of the body; engaging philosophically with concepts of health and illness; exploring broader notions of health, well-being and human flourishing; valuing subjective perspectives on the experience of illness; engaging non-medical practitioners as research partners; recognizing alternative spaces of healthcare; and challenging dominant epistemological frameworks through new methodologies (Kearns and Collins 2010; Kearns and Moon 2002). However, and even more instructively for the medical humanities, these debates within the geographies of medicine and health have developed further to reconsider the distancing from “the medical” within a critical social theory, to blur this once-cherished distinction and recover new encounters between “the medical,” “health” and critical theory within geography (Parr 2002).

## Critical engagements: medicine and health in contemporary biopolitics

In a review of the field at the turn of the Millennium, Parr (2002) defined two trends within “medical geography.” The first she argued was premised on “a stark retreat from things medical, resulting in “geographies of health” solely concerned with “healthy” spatialities which somehow exist beyond explicitly biomedical categorizations, treatments and practices” (241). As Kearns and Gesler argue, the vision here is one of “a more progressive medical geography – a medical geography released from the shadow of medicine and reinvented as geographies of health and healing” (1998, 3). However, Parr draws attention to a second approach which does not aim to “‘do away with’ the medical, but [continues] to engage with it, albeit in a more critical capacity than has been the case previously within the subdiscipline” (241). The key issue raised by medical/health geographers then, is not simply about the expansiveness of the field of enquiry, but of the field’s orientation to what might be meant by “the medical.” Although aiming to challenge the dominance of “the medical” in medical humanities, by arguing principally for an expansionist account, Crawford et al’s (2010) account of the health humanities leaves the power associated with “the medical” unchallenged, allows those claiming to speak from a “medical” position to continue to claim an authority which similarly remains unchallenged, and seems to preclude the possibility of grappling with the phenomena of “medicalization” or the expansion of a medical gaze (http://www.healthhumanities.org/ or http://en.wikipedia.org/wiki/Health_humanities).

Within geography, several strands of work have demonstrated engagements among critical theory, biomedical concerns and the relationalities of health, space and place. The central concern of critical theory with the ways in which power and politics interact with bodies, biology, health and life itself challenges us to consider how new knowledge is shaped, how it influences our understandings of ourselves and how such framings enable a seamless translation into response and responsibility. Geographers have examined this inseparable connection, variously captured by the terms biopower, biopolitics or biosociality (Foucault 1977; 1991; Rabinow and Rose 2006; Rose 2001), with particular emphasis on the expression of the complex interactions involved in biopolitics as it relates to medicine and health at particular times and in particular places. For example, McPhail (2009) discusses how concerns about obesity in early cold war Canada reflected broader political concerns about the “solidity” of the nation’s borders and of nuclear family life in the wake of increased immigration and the threat of war. In a similar vein, Craddock (1999) explores the co-production of race, place and pathology in relation to smallpox epidemics in 19th century San Francisco illustrating how a political anatomy of the Chinese body read disease and depravity into its fundamental structure and simultaneously stigmatized both China Town and Chinese bodies.

A renewed emphasis in biology on molecular life has been accompanied by a new social understanding of our biological selves, a “molecularization of life” that shifts our political engagements with our biological selves. Braun (2007) identified two distinct orientations within work on the matter of life itself, one informed by a Foucault’s notion of governmentality and one informed by the threats of infectious disease. The first of these has been dominant across a range of social science research drawing on Foucault to offer a critical engagement with much of contemporary health policy and medical practice. Such research argues that contemporary forms of governmentality characterise the individual as an autonomous agent holding responsibility for his or her own health and well-being. At the same time, choice is directed and governed through a host of measurements and assessments of outcomes (Miller and Rose 2008). Researchers have challenged the ways in which bodies are governed through such calculative techniques and technologies in relation to the policy attention given to obesity (Evans and Colls 2009) and alcohol (Jayne et al. 2011); the ways in which different bodies are positioned as more, or less, capable of acting responsibly in contemporary public health (Colls and Evans 2008; Evans 2010; Evans et al. 2011) and the ways in which particular forms of bodily matter (such as fat) come to *matter* (Colls 2007).

There is a strand of inquiry driven by concerns of biosecurity which positions individual bodies as vulnerable to flows of molecular hazards that are neither visible, predictable nor initially framed as amenable to individual control. Braun’s particular argument (2007) is that the conventional focus within either medical or health geographies means that social scientists and geographers in particular have paid insufficient critical attention to the ways that threats to biosecurity may reconfigure contemporary biopolitics or the ways such relations may interrelate with the concerns of governmentality. However, recent research has begun to redress a “governmentality” bias in critical engagements. Sparke and Anguelov (2012) explore how the framing of and responses to the pandemic in 2009 of H1N1 virus, popularly known as “swine-flu,” reflected and reinforced existing multiple inequalities. These are seen in the positioning of blame for the outbreak onto poor countries and poor people, in the calculation and management of risk, in access to treatment both globally and within any one nation-state, and in the ways global processes of neoliberalisation help produce the emergence of new and virulent flu viruses. The authors unpack the ways in which global concerns of biosecurity are woven together with a contemporary emphasis on individualized responsibility of risk and response. In a similar vein, Mansfield (2012a, b, c) examines the framings of environmental contamination in fish products which can affect foetal neurodevelopment. Mansfield’s critical engagement with a policy response that advises against consumption reveals a form of gendered and racial biopolitics. The importance of this work is that it draws together highly biomedical and biochemical concerns, concerns conventionally located under “the medical,” with the interests of a critical theory, typically located under “health” through concerns with the biopolitics of health, life or well-being. Moreover, this welding of the medical, health and critical theory explicitly provokes new engagements with space and time in relation to material bodies and environment-body interactions (Guthman and Mansfield 2012).

## Medical, health and the radical potential of the medical humanities

What lessons might the medical humanities take from this exploration of “the medical” and “health” in the affiliated fields of the social sciences and in geography specifically? In part, the answer depends on how we understand the role for the medical humanities more widely, and here there are of course a range of viewpoints. Should medical humanities retain their focus on medical education, adopt the position of medicine’s “supportive friend” (Brody 2011), become a disciplinarily “disruptive teenager” (Macnaughton 2011), or seek something else altogether? In particular, we ask, is there radical potential for the medical humanities? As our discussion of medical/health geography has made clear, our key argument is for a closer engagement with critical and cultural theory. To conclude this paper, we discuss (i) intersubjective and relational approaches to well-being, and (ii) activists’ use of the arts and creative practice to disrupt medical definitions, categories and practices and to campaign for social justice, as two examples of the strength of such an engagement.

Recent work within the critical social sciences has argued for a re-configuration of the subject beyond a neoliberally-bounded model to one that recognizes intersubjectivity and intercorporeality. This move extends the existing critique of “individualistic liberalism,” which asserts the rights of already constituted subjects by instead emphasizing the “relational constructedness of things” (subjectivities, bodies, spaces, etc) (Massey 2005, 10). In short, this is an approach to “the body” that focuses on “the connections that bind us together” (Lawson 2007, 4), and it reconceptualizes space and “the social” in terms of interrelations, multiplicity, heterogeneity and flux (Massey 2005). Within such work, therefore, “the body” is not understood as a bounded, singular body-subject but is instead involved in a “constant, ongoing process of connection with other human, non-human, past, present and future bodies” (Evans et al. 2011, 324). Health, then, is reconceptualized as something that is produced through the relations between bodies rather than as something that a body is or is not.

While the medical humanities has done a lot to challenge dominant medical perspectives, it seldom if ever ventures beyond a neoliberal, humanist notion of the individual body-subject and associated conceptualizations of responsibility, rights, and risk management to really explore alternative “collective” and “relational” approaches to “flourishing.” As well as learning from critical theory, here the medical humanities can also learn much from the practice of arts and health. By contrast to a medicalized arts therapy, which tends to focus on the internal dimensions of individuals’ trajectories of ill-health, the participatory ethos of arts and health engages with the social or collective dimensions and determinants of health to foster personal and community well-being, explicitly conceptualizing these as inextricably interwoven (Atkinson and Robson 2012; Atkinson and Rubidge 2013; White 2009). This is also a feature of arts and health practice that is addressed to specific types of communities, or experiences of ill-health, where, as Parr describes, “a distinctive theme is a sense of shared illness experience” (2006, 158). Agencies providing participatory arts and health activities constantly face the challenge of negotiating the demands of funders informed by the dominant model of a neoliberal subject whilst maintaining their own ethos of collective and relational care for well-being (Swan and Atkinson 2012). Without detracting from the importance of individual experience or silencing dissenting voices, such a collective approach questions the desirability of “personalized care” that separates the “ill” individual from the communities and spaces within which he or she lives. The argument for greater attention to collective, relational and situated understandings of differentiated experiences of health and ill-health is timely within the medical humanities as major political and policy changes are afoot within the contemporary provision of health care. For example, our understandings of personal narratives of ill-health experiences, something constituting a major area of research within the medical humanities, disclose as much about the politics of ill-health experiences as the immediate health care needs if interpreted as intersubjective and situated in their construction (Atkinson and Rubinelli 2012). Such engagements within the medical humanities disrupt the view of the individual as always and necessarily a bounded subject and challenges the spatio-temporalities in dominant concepts of recovery and therapy by recognizing that well-being is always in flux and allowing “space for differentiated self-development.”

Healing, well-being, happiness, wonder, beauty and empathy are important concepts in the medical humanities literature, but we would also argue that the medical humanities has a role to play in exploring the value and productivity of emotions seen as ‘negative’ and looking beyond the classics of the Western canon to engage with forms of creative practice that may unsettle and disrupt the ways in which particular bodies and subjects are defined as healthy or not. Again, there is no shortage of medical humanities work on illness narrative and the subjective experience of illness, but with little critique of the wider political climate within which such work emerges, it is dominated by positivity and praise of heroic survivorship (Bartels 2009; Ehrenreich 2009; King 2006) and by the failure to recognize its own cultural and historical specificity (Hooker and Noonan 2011). The medical humanities have also accorded negligible attention to the art, arguments and activities of activist movements. Mad pride events, for example, feature cogent and carnivalesque critiques of what counts as “mental health”; fat activist groups such as ‘the Chubsters,’ based in the UK describing themselves as ‘a vicious girl gang’ and who aim to disrupt medicalized notions of fatness through performances which emphasize anger, rage and humour (http://www.chubstergang.com/). It is important to emphasize that the approach we are advocating is not simply about fitting ‘pride’ movements within a model of ‘recovery’ – the role of such arts-lead activism is not to help those whose bodies/minds do not fit or feel comfortable with their place in the world (although this is part of it); rather it is to challenge the ways in which such bodies are approached within medicine and health and in the wider culture, and to recognize anger as a productive force (Ahmed 2010, 108).

In conclusion, we are calling for the medical/health humanities to take seriously the challenges of critical and cultural theory, community-based arts-in-health, and the counter-cultural creative practices and strategies of activist movements. We are calling, in other words, for an evolution to a *critical* medical humanities which would enhance the intellectual as well as the “real-world” impact of our field’s interrogations of medicine, health and illness.
